# P-210. Real-World Utilization and Cost of Fidaxomicin versus Oral Vancomycin: A Comparative Analysis of Outpatient Medicaid Prescription Reimbursements

**DOI:** 10.1093/ofid/ofae631.414

**Published:** 2025-01-29

**Authors:** Jessica C O’Neil, Ebbing Lautenbach

**Affiliations:** University of Pennsylvania, Philadelphia, Pennsylvania; University of Pennsylvania, Philadelphia, Pennsylvania

## Abstract

**Background:**

Clostridioides Difficile Infection (CDI) is a leading cause of infectious diarrhea in the United States. In 2017, the Infectious Diseases Society of America (IDSA) and Society of Healthcare Epidemiology of America (SHEA) joint guidelines added fidaxomicin (FX) as a first line therapy for initial and first recurrence of non-fulminant CDI alongside oral vancomycin (OV). Under a 2021 guideline revision FX became the preferred therapy based on lower CDI recurrence among those treated with FX. Multiple analyses have attempted to predict the economic impact of increased FX use but have yielded conflicting results. The use of real-world data on actual cost and utilization has been limited.

**Figure 1:**
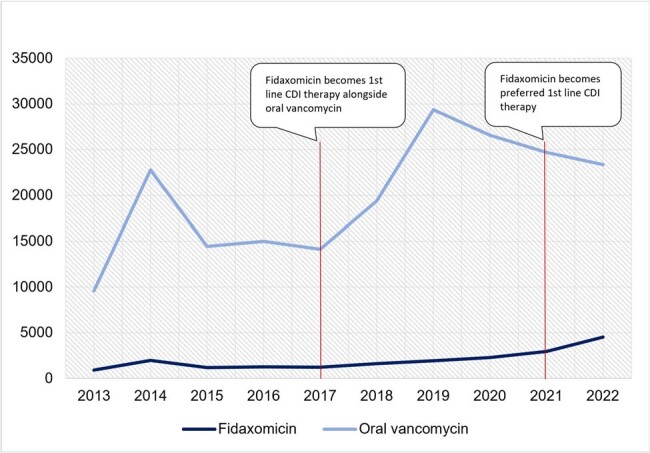
Total Annual Outpatient Medicaid CDI Therapy Prescription Count CDI: Clostridioides Difficile Infection

**Methods:**

We conducted a retrospective analysis of utilization and cost of FX and OV between January 1^st^, 2013 and December 31^st^, 2022 in the Medicaid State Drug Utilization Database (SDUD). The SDUD contains covered outpatient medications excluding those reimbursed under bundled services. Prescriptions for OV were distinguished from other vancomycin formulations using national drug codes. Utilization was estimated by the number of prescriptions reimbursed. Total cost was calculated as the sum of Medicaid and non-Medicaid reimbursement. We approximated the annual per prescription cost (APPC) as the quotient of total cost and prescription count.

**Figure 2:**
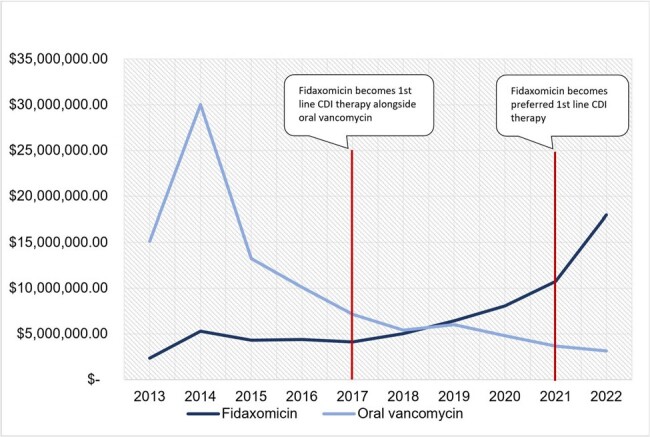
Total Annual Outpatient Medicaid CDI Therapy Prescription Cost CDI: Clostridioides Difficile Infection

**Results:**

Following the 2017 guidelines the average annual FX prescription count (Figure 1) increased by 32% compared to a 2013-2016 baseline. After the 2021 guideline revision, average annual FX prescription count grew by 113% as compared to a 2017-2020 baseline. The total annual cost of FX (Figure 2) surpassed the total annual cost of OV in 2019 with FX reaching $17,984,998 in 2022 as compared to $3,135,523 for OV. Throughout the study period the FX APPC (Figure 3) was substantially greater ($2,614-$3,956) than the OV APPC ($134-$1,579).

**Figure 3:**
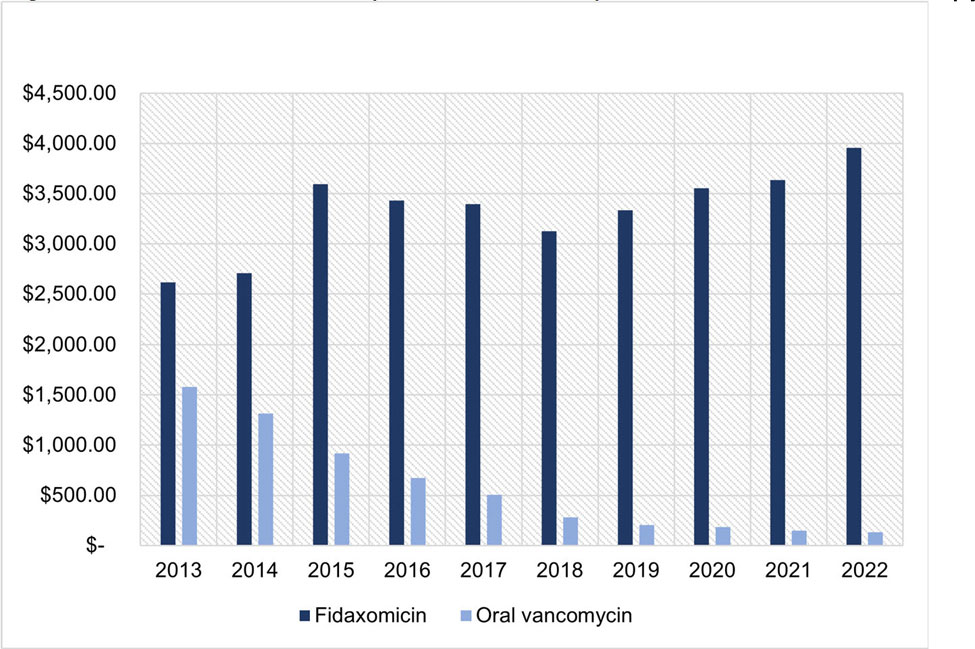
Annual Per Prescription Cost of Outpatient Medicaid CDI Therapy CDI: Clostridioides Difficile Infection

**Conclusion:**

Among Medicaid beneficiaries, outpatient FX use increased in time periods following the 2017 and 2021 guideline changes. While FX use was significantly lower than OV from 2013-2022, FX total cost and APPC far outweighed that of OV. Further analysis is necessary to determine the real-world cost effectiveness of FX, but our results highlight the budgetary impact associated with these IDSA/SHEA guideline changes.

**Disclosures:**

**All Authors**: No reported disclosures

